# Spectroscopic Investigation of the Interaction of the Anticancer Drug Mitoxantrone with Sodium Taurodeoxycholate (NaTDC) and Sodium Taurocholate (NaTC) Bile Salts

**DOI:** 10.3390/molecules22071079

**Published:** 2017-06-28

**Authors:** Mirela Enache, Ana Maria Toader, Victoria Neacsu, Gabriela Ionita, Madalin I. Enache

**Affiliations:** 1 Institute of Physical Chemistry Ilie Murgulescu, Romanian Academy, Splaiul Independentei 202, Bucharest 060021, Romania; ancutatoader@yahoo.fr (A.M.T.); neacsu_victoria@yahoo.com (V.N.); ige@chimfiz.icf.ro (G.I.); 2 Institute of Biology, Romanian Academy, Splaiul Independentei 296, Bucharest 060031, Romania; madalin.enache@ibiol.ro

**Keywords:** mitoxantrone, bile salts, binding constant, partition coefficient, UV-Vis absorption spectroscopy, electron paramagnetic resonance spectroscopy

## Abstract

The focus of the present work was to investigate the interaction of the anticancer drug mitoxantrone with two bile salts, sodium taurodeoxycholate (NaTDC) and sodium taurocholate (NaTC). Ultraviolet-visible (UV-Vis) absorption and electron paramagnetic resonance (EPR) spectroscopy were used to quantify the interaction and to obtain information on the location of mitoxantrone in bile salt micelles. The presence of submicellar concentrations of both bile salts induces mitoxantrone aggregation and the extent of drug aggregation in NaTDC is higher than in NaTC. For micellar bile salts concentrations, mitoxantrone monomers are entrapped in the micellar core. Binding constants, micelle/water partition coefficients and the corresponding thermodynamic parameters for binding and partitioning processes were estimated using the changes in monomer absorbance in the presence of bile salts. Binding interaction of mitoxantrone is stronger for NaTDC than NaTC micelles, whereas partitioning efficiency is higher for NaTC micelles for all investigated temperatures. Thermodynamic parameters indicate that both binding and partitioning processes are spontaneous and entropy controlled. The spectral behavior and thermodynamic parameters indicate distinct types of mitoxantrone interaction with NaTDC and NaTC micelles supported by the differences in nature and structure of bile salts micelles.

## 1. Introduction

Bile salts belong to the class of natural steroids and are synthesized in the liver and stored in the gallbladder. In addition to their biological role in digestion and gallstone formation, bile salts have also received much attention as drug delivery systems and are used as membrane penetration enhancers in drug formulations [1,2,3,4,5]. The general structure of bile salts is very different from that of common surfactants. Bile salts have a rigid steroidal skeleton consisting of four rings, out of which three rings are six membered and one is five membered ring. Hydrophilic groups are attached to the hydrophobic ring system: between one and three hydroxyl groups and one acidic group. Human bile salts differ in the number, position and stereochemistry of the hydroxyl groups as well as in the conjugated aminoacid, either taurine or glycine. The hydrophilic part of the bile salts is represented by the concave side of the rigid steroid ring system bearing the hydroxyl groups, while the hydrophobic part is represented by the convex side of the steroidal skeleton. As a consequence of their rigid facial like structure characterized by a weak separation between the hydrophilic and hydrophobic regions, the micellar aggregation of bile salts is driven by both, the hydrophobic effect and hydrogen binding [6,7]. Due to the presence of different binding sites, bile salt aggregates can carry both hydrophilic and hydrophobic drug molecules depending of the structure and size of the guest molecules [8,9,10,11].

The aggregation feature and the shape of the micelles of bile salts are different from those of conventional surfactants. Several models have been proposed to describe the unusual micellization behavior of bile salts. The most accepted model known as Small’s model is a two-step model with the formation of primary and secondary micelles [12]. In the first step, around the critical micellar concentration (CMC), the bile salt monomers start to aggregate to form primary micelles containing 2–10 monomers through hydrophobic interactions between the hydrophobic sides of the monomers. In a second step, at higher concentration, the primary aggregates further interact to form larger secondary micelles by hydrogen bonding among the hydroxyl groups located on the surface of primary micelles. This particular micellization behavior determines much lower values of CMC and aggregation number of bile salts than in the case of usual surfactants with a linear structure.

**Figure 1:**
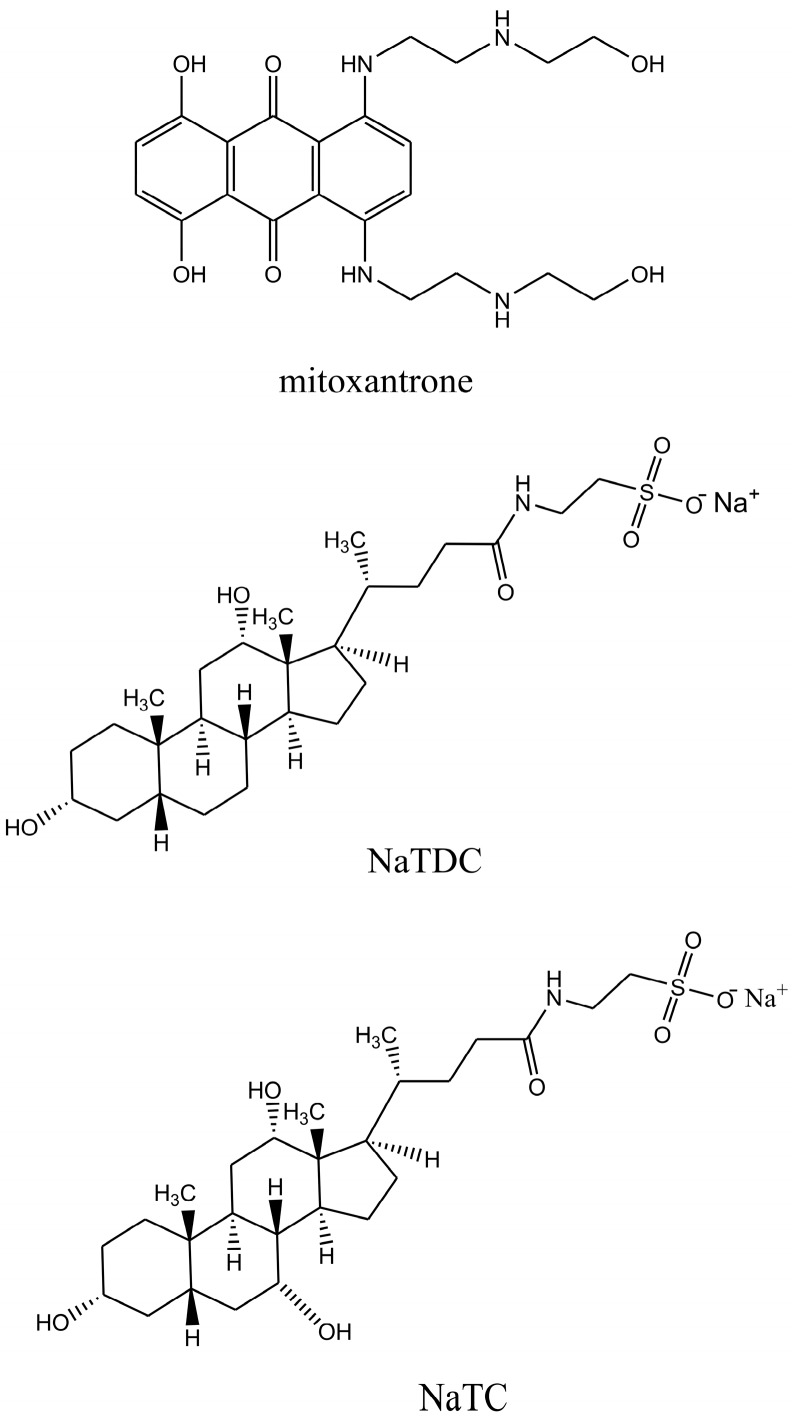
Chemical structures of mitoxantrone, NaTDC and NaTC.

Mitoxantrone (1,4-dihydroxy-5,8-bis[2-(2-hydroxyethylamino)ethylamino]-anthracene-9,10-dione) is a synthetic anthracenedione chemotherapeutic drug which has shown significant clinical efficiency in the treatment of breast cancer, lymphoma, acute leukemia and multiple sclerosis [13,14,15,16]. Mitoxantrone contains a hydrophobic planar chromophore substituted with two side chains containing nitrogen, positively charged at physiological pH (Figure 1). The anticancer activity of mitoxantrone is mainly attributed to its interaction with nuclear DNA by intercalation of the planar anthraquinone ring between DNA base pairs, whereas the side groups interact electrostatically with the negatively charged phosphate groups of DNA [17,18,19,20,21,22]. Mitoxantrone also inhibits the activity of topoisomerase II, an enzyme involved in the repair of damaged DNA [23,24].

If the initial purpose of the synthesis of mitoxantrone was to reduce the cardiotoxic side effects of anthracyclines, further studies extensively reported the cardiotoxicity occurred during the mitoxantrone therapy [25,26]. Mitoxantrone is also a substrate for efflux mediated by members of ATP binding cassette (ABC) transporters, which results in the resistance of cancer cells to mitoxantrone treatment thus limiting its clinical use [27]. 

A drug molecule has to pass through the cellular and nuclear membranes before to reach their target inside the cancer cells. Because the biological membranes represent complex multicomponent structures, the surfactant micelles with much less complexity have been used as model systems for biomembranes aiming to investigate the interactions of different drug molecules with biological membranes [28,29,30,31,32,33,34]. The surfactant micelles are also useful in drug delivery as they ensure the transport to specific sites of action, minimize drug degradation and loss, prevent harmful side effects and increase drug bioavailability [35,36].

In our previous papers, we reported a physico-chemical investigation of interactions between mitoxantrone and anionic, cationic and non-ionic micelles by employing spectral and electrochemical measurements [37,38,39,40]. In continuation of our interest in anticancer drug mitoxantrone and its interaction with biomimicking structures, in the present study the mitoxantrone-bile salts system has been investigated by UV-Vis absorption and EPR spectroscopy. Sodium taurodeoxycholate (NaTDC) and sodium taurocholate (NaTC) bile salts used in the present study have the same head group (–CO–NH–CH_2_–SO_3_^−^) but NaTC contains one hydroxyl group more in the hydrophilic surface and this difference makes NaTC more hydrophilic than NaTDC (Figure 1). The changes in the absorption spectra of mitoxantrone in the presence of bile salts were used to calculate the binding constants, stoichiometric ratios, partition coefficients of mitoxantrone between aqueous and micellar phases and the corresponding thermodynamic parameters for the above two processes. These parameters predict the strength of binding between mitoxantrone and bile salts. EPR spectroscopy can provide useful information on the topology and dynamic aspects of systems generated by aggregation process of natural or synthetic surfactants [41,42]. The spin probe method is often used in investigating diamagnetic systems emerging from self-assemblies through non-covalent interaction of their constituents. The spin probe method has been applied so far in studying surfactant solutions, nanostructure materials, and the accessibility of low molecular mass molecules to porous materials, self-assembled monolayers or on gold nanoparticles [41]. In this study, the amphiphilic spin probes from the family of *n*-doxyl-stearates were chosen to provide information on the self-assembly of NaTDC and NATC bile salts. These spin probes are characterized by sensitivity of their EPR spectra to restricted motion when are incorporated in micellar solutions. This general behavior of doxyl spin probes is determined by the fact that paramagnetic moiety is rigidly attached to the alkyl chain.

## 2. Results and Discussion

### 2.1. UV-Vis Absorption Studies

The absorption spectra of mitoxantrone—bile salts solutions containing different NaTDC or NaTC concentrations were recorded at different temperatures in order to investigate the molecular interactions between mitoxantrone and bile salts molecules.

**Figure 2:**
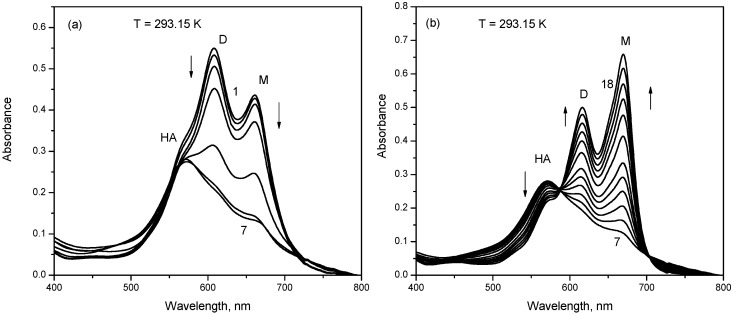
Absorption spectra of mitoxantrone (2.80 × 10^−5^ M) in phosphate buffer pH 7.4 and 293.15 K temperature, in the presence of different sodium taurodeoxycholate (NaTDC) concentrations: (**a**) [NaTDC] = 0 (spectrum 1)–1.88 × 10^−3^ M (spectrum 7); (**b**) [NaTDC] = 1.88 × 10^−3^ M (spectrum 7)–3.22 × 10^−2^ M (spectrum 18). The arrows indicate the decrease or increase of monomer (M), dimer (D) and higher aggregates (HA) absorption bands.

In the absence of bile salts, the visible absorption spectrum of mitoxantrone in phosphate buffer pH 7.4 shows two absorption bands at 660 and 610 nm, and a shoulder at about 570 nm. Based on the dependence of the absorption spectrum of mitoxantrone on concentration, the band at 660 nm was attributed to the monomer (M), the band at 610 nm to the dimer (D) and the band around 560 nm to the formation of the higher aggregates (HA) of the drug [43]. The absorption spectra of 2.80 × 10^−5^ M mitoxantrone obtained in the presence of various NaTDC concentrations at 293.15 K temperature are shown in Figure 2, where it can be observed that the spectral absorption behavior of mitoxantrone is dependent on the NaTDC concentration. On gradual addition of NaTDC, a hypochromic effect is observed on both monomer and dimer bands (Figure 2a). When the concentration of NaTDC is about 1.88 × 10^−3^ M, the monomer and dimer bands practically disappear and the band at 570 nm corresponding to higher aggregates is outlined very clearly (spectrum 7 in Figure 2a).

**Figure 3:**
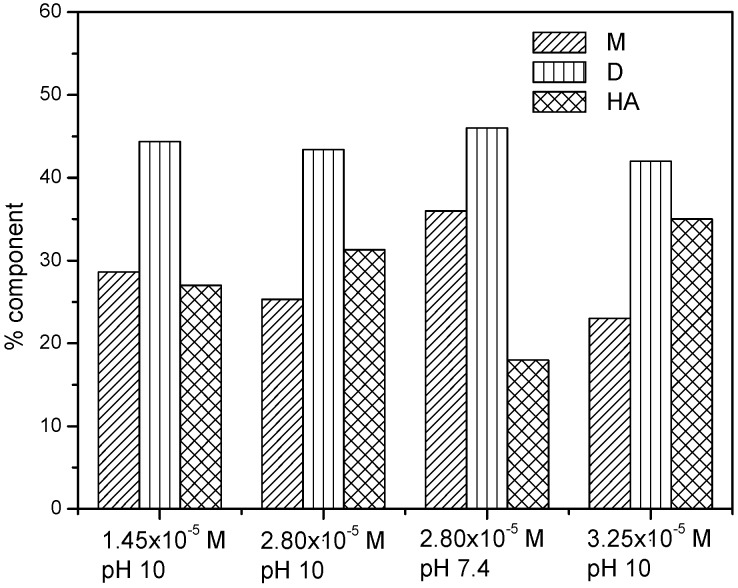
Evolution of M, D and HA band areas in deconvoluted spectra for different mitoxantrone concentrations in carbonate buffer pH 10 and phosphate buffer pH 7.4.

A further increase of NaTDC concentration determines the increase of absorbance of both 610 nm and 660 nm bands but the absorbance band at 660 nm corresponding to the drug monomer becomes predominant (spectrum 18 in Figure 2b) and even more pronounced than that in the absence of NaTDC (spectrum 1 in Figure 2a). At the same time, the absorbance of 570 nm band corresponding to the higher aggregates of the drug decreases. The CMC of NaTDC in 0.1 M salt solution is 2 mM [44]. Mitoxantrone has two ionizable amines on lateral chains with p*K*_a_ values of 8.3–8.6 [45]. Therefore, under the experimental conditions of pH 7.4, mitoxantrone is present as bivalent cation due to the two protonated nitrogen atoms from lateral chains. At the same time, both NaTDC and NaTC molecules exist as anions (p*K*_a_ less than 2). Consequently, the strong molecular interaction of mitoxantrone with NaTDC observed at concentrations lower than CMC can be assigned to the formation of mitoxantrone-NaTDC complex due to the electrostatic interaction between the cationic drug molecules and the anionic bile salt monomers. In our previous studies, we have investigated the interaction of mitoxantrone with anionic (sodium dodecyl sulfate, SDS) [37], cationic (cetyltrimethylammonium bromide, CTAB) [38] and non-ionic (Triton X-100, Tween-20, Tween-80) [39] surfactants. Comparing all these spectral results, we observe the same spectral behavior of mitoxantrone in the presence of NaTDC and SDS (decrease of both monomer and dimer bands for surfactant concentration lower than CMC). At the same time, this spectral behavior was not observed in the presence of cationic and non-ionic surfactants indicating the electrostatic nature of the interaction between mitoxantrone and the anionic surfactants (SDS and NaTDC). The decrease of absorbance observed for NaTDC concentrations lower than CMC may be explained by the neutralization of mitoxantrone positive charges by the bile salt anions and this electrostatic interaction decreases the repulsive forces between the positively charged drug molecules. Consequently, this electrostatic interaction may induce premicellar surfactant aggregation and favors the aggregation of the drug molecules [46,47,48,49]. The presence of mitoxantrone–NaTDC complexes at bile salt concentrations lower than CMC induces a strong aggregation of mitoxantrone molecules and the bands of monomer and dimer disappears and higher aggregates are formed, as is indicated by the spectrum 7 in Figure 2a. While in the case of SDS, the presence of mitoxantrone-SDS complexes induces the dimerization of mitoxantrone [37], the presence of mitoxantrone-NaTDC complexes leads to the formation of higher aggregates. Our previous investigation showed that mitoxantrone forms dimers in phosphate buffer pH 7.4 at concentrations higher than 1.00 × 10^−6^ M, and the dimerization process is dependent on pH, the dimerization constant increasing with the increase of pH [43]. In order to get a deeper insight into the aggregation behavior of mitoxantrone, we recorded the absorption spectra of drug in carbonate buffer pH 10 at different concentrations (data not shown). It was observed that the shape of the absorption spectrum is dependent on concentration, similar with the behavior in phosphate buffer pH 7.4 but the shoulder at about 570 nm assigned to the higher aggregates of the drug is more evident. Also, the shape of the spectrum does not change with temperature, but the monomer and dimer absorption bands increases on expanse of higher aggregates, suggesting the dissociation process of these aggregates with increasing temperatures. In order to follow the evolution of monomer, dimer and higher aggregates bands of mitoxantrone in different experimental conditions, the absorption spectra were deconvoluted in elementary bands. Figure 3 shows the variation of monomer, dimer and higher aggregate components with mitoxantrone concentration and pH 10 and 7.4.

It can be observed that in carbonate buffer pH 10, the dimer component is almost constant for increasing mitoxantrone concentrations but the higher aggregates component increases on expense of monomers. Also, for the same mitoxantrone concentration, the dimer component is almost constant for pH 7.4 and 10 but the higher aggregates component is 1.7 times higher in pH 10, indicating mitoxantrone aggregation in basic medium. 

**Figure 4:**
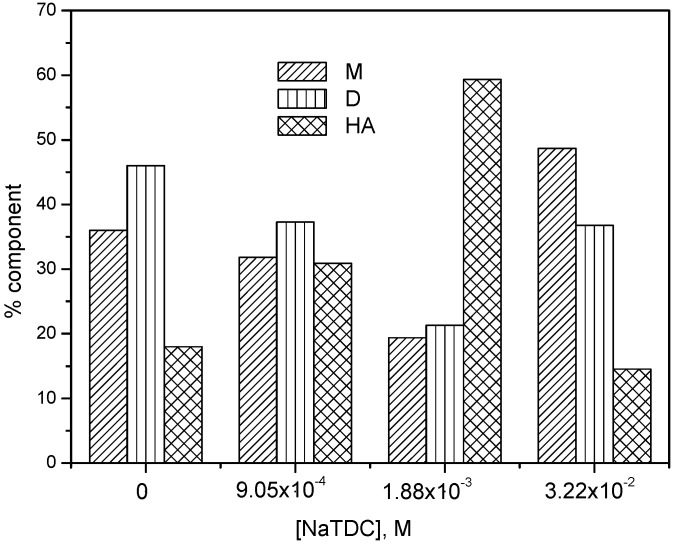
The percent of component band areas in deconvoluted spectra for M, D and HA for 2.80 × 10^−5^ M mitoxantrone in phosphate buffer pH 7.4 in the presence of different NaTDC concentrations.

The variation of monomer, dimer and higher aggregate components with NaTDC concentration is shown in Figure 4.

It can be observed that for increasing NaTDC concentrations up to 1.88 × 10^−3^ M the higher aggregates component increases drastically (about 3.3 times) on the expense of monomer and dimer components, indicating a strong aggregation of mitoxantrone induced by the presence of mitoxantrone-NaTDC premicellar aggregates. 

When the NaTDC concentration is higher than CMC, both monomer and dimer absorbance bands increase (the absorbance band of drug monomer is predominant) and the higher aggregates absorbance maxima decreases (Figure 2b). As the NaTDC concentration increases and the micelles start to form, the mitoxantrone aggregation is reduced and mitoxantrone is solubilized in the micelles in its monomer form. The presence of NaTDC micelles yields two isosbestic points at 587 and 705 nm in absorption spectra of mitoxantrone. Moreover, at NaTDC concentrations above CMC a red shift of both absorbance maxima can be observed (616 and 669 nm, respectively). For NaTDC concentrations higher than CMC, the monomer and dimer components increases and the higher aggregate component decreases, indicating the dissociation of higher aggregates into dimers and monomers due to the interaction of mitoxantrone with NaTDC (Figure 4).

**Figure 5:**
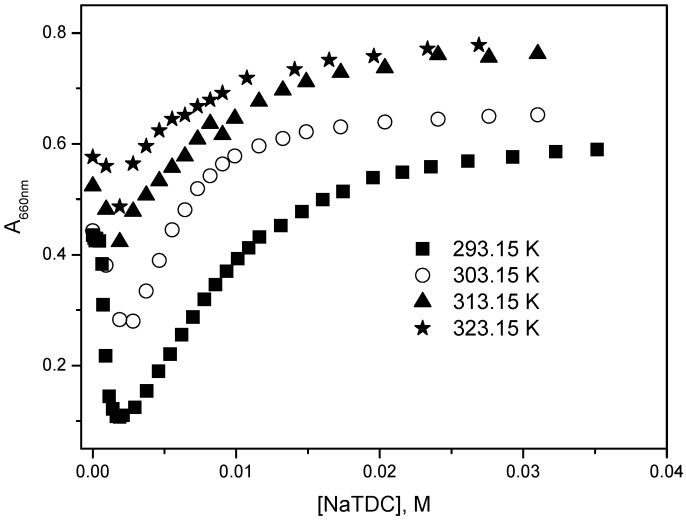
Variation of mitoxantrone absorbance at 660 nm with NaTDC concentration at different temperatures.

The variation of monomer absorbance as a function of NaTDC concentration at different temperatures (Figure 5) indicates two distinct processes: process I (the decrease of absorbance) in premicellar range of NaTDC concentrations, and process II (the increase of absorbance) at NaTDC concentrations higher than CMC. The two processes are similar to those observed in the case of the interaction of mitoxantrone with SDS [37]. For NaTDC concentrations higher than about 2.00 × 10^−2^ M, the monomer absorbance attains almost a constant value for all investigated temperatures indicating that the mitoxantrone monomers are completely solubilized in the NaTDC micelles. Also, it can be observed that for increasing temperatures the process I is less evident. As in the case of SDS [37], the decrease of monomer absorbance (process I) may be explained by the neutralization of mitoxantrone positive charges by the bile salt anions that eliminates the repulsive forces between drug molecules and favors the drug aggregation; the increase in monomer absorbance (process II) is due to the interaction of mitoxantrone monomers with NaTDC micelles.

**Figure 6:**
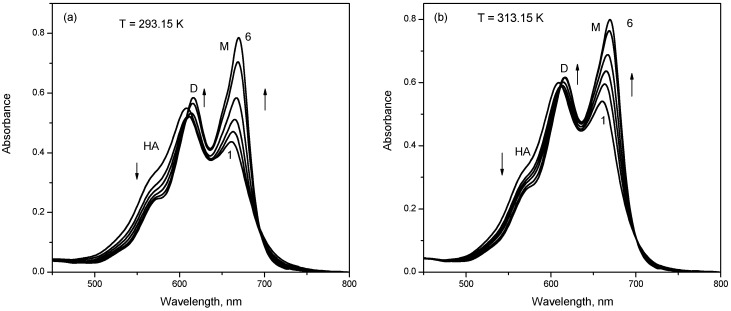
Absorption spectra of mitoxantrone (2.80 × 10^−5^ M) in phosphate buffer pH 7.4 in the absence (spectrum 1) and the presence of different NaTC concentrations ([NaTC] = 4.62 × 10^−4^ M (spectrum 2); 1.21 × 10^−2^ M (spectrum 6) and at two different temperatures ((**a**), 293.15 K; (**b**), 313.15 K). The arrows indicate the decrease or increase of M, D and HA absorption bands.

Figure 6 presents the absorption spectrum of mitoxantrone in the presence of increased concentrations of NaTC for two different temperatures. In contrast with NaTDC, it can be observed that for NaTC concentrations lower than CMC the absorbance of both 610 nm and 660 nm bands increases and the monomer absorbance becomes predominant. At the same time, the absorbance of 560 nm band corresponding to the higher aggregates of the drug decreases.

**Figure 7:**
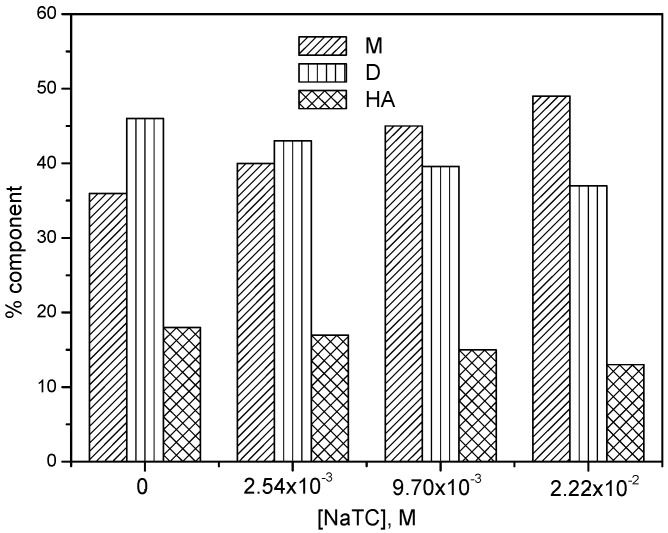
The percent of component band areas in deconvoluted spectra for M, D and HA for 2.80 × 10^−5^ M mitoxantrone in phosphate buffer pH 7.4 in the presence of different NaTC concentrations.

The variation of monomer, dimer and higher aggregate components with NaTC concentration obtained from deconvolution of the spectra is shown in Figure 7. As against NaTDC, it can be observed that the monomer component increases on the expense of dimer and higher aggregates as NaTC concentration increases.

**Figure 8:**
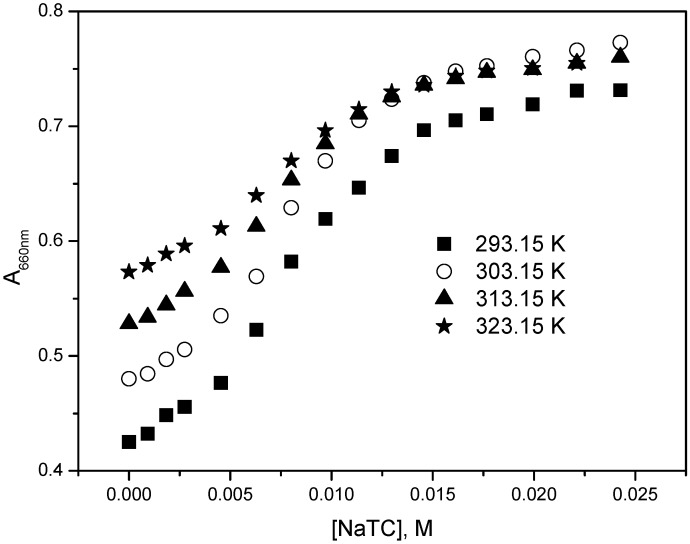
Variation of mitoxantrone absorbance at 660 nm with NaTC concentration at different temperatures.

The variation of the monomer absorbance of mitoxantrone as a function of NaTC concentration is shown in Figure 8 for different temperatures. It can be observed that the monomer absorbance increases nearly linear with the concentration of NaTC until around 5 mM concentration which is the CMC of NaTC [44]. For higher NaTC concentration, the monomer absorbance becomes almost constant indicating the complete solubilization of mitoxantrone monomers in NaTC micelles. Also, the presence of NaTC induces a red shift of both monomer and dimer absorbance maxima of the drug (617 and 670 nm, respectively). The increase in the monomer absorbance and the red shift of absorption maxima, even for submicellar NaTC concentrations, indicate a strong electrostatic interaction between anionic NaTC and cationic mitoxantrone monomers.

**Figure 9:**
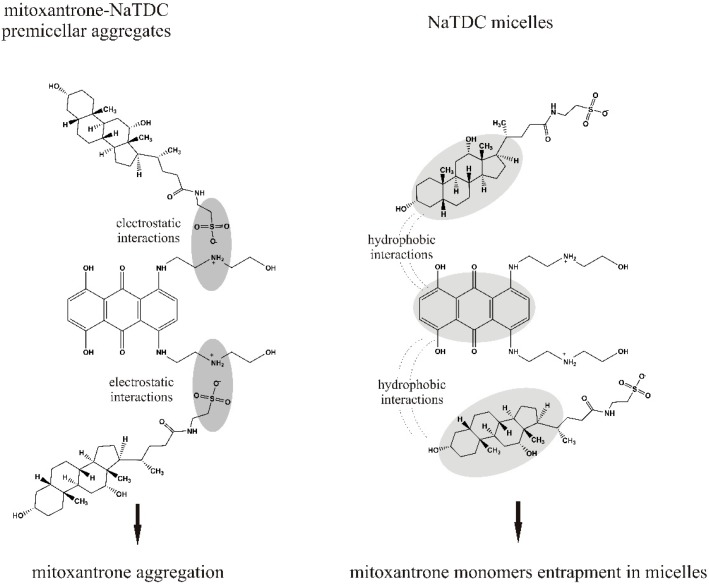
Schematic illustration of the interactions between mitoxantrone and NaTDC.

By comparing the interaction of mitoxantrone with NaTDC and NaTC, we can say that the spectral behavior dependents on the type of bile salt. NaTDC and NaTC have the same head group (–CO–NH–CH_2_–SO_3_^−^) but differ in the number of hydroxyl groups in the hydrophilic surface: NaTDC has two hydroxyl groups while NaTC contains three hydroxyl groups. Also, the hydrophobicity indices indicate that NaTDC is more hydrophobic (hydrophobicity index 0.59) than NaTC (hydrophobicity index 0) [8]. For bile salt concentrations lower than CMC, the first step in the interaction of mitoxantrone with NaTDC and NaTC is the formation of mitoxantrone-NaTDC (NaTC) complexes by electrostatic interaction between positively charged mitoxantrone molecules and negatively charged bile salt monomers (Figure 9).

When the mitoxantrone-bile salt complexes are formed, the charges on both mitoxantrone and bile salt monomers are neutralized leading to the suppression of electrostatic repulsion between positively charged drug molecules and aggregation of mitoxantrone occurs. In the case of NaTDC, the formation of mitoxantrone-NaTDC aggregates induces a higher aggregation of mitoxantrone (the higher aggregates are the major component in solution) compared to NaTC when monomer and dimer are the major components in solution. Experiments performed in pH 10 carbonate buffer, when mitoxantrone molecules are uncharged due to deprotonation of NH_3_^+^ groups of the side chains, indicated that the dimer component is almost constant for increasing mitoxantrone concentrations but the higher aggregate component increases at the expense of monomers. However, the formation of mitoxantrone-NaTDC aggregates induces a higher drug aggregation than basic medium, most likely this behaviour being the result of concerted electrostatic interactions and hydrophobic interactions between the aromatic chromophore of mitoxantrone and the hydrophobic surface of NaTDC. Also, the presence of mitoxantrone-NaTDC aggregates leads to a higher aggregation of mitoxantrone than in the case of anionic surfactant SDS [37]. In the case of the interaction of Nile Blue A dye with bile salts, the results indicated that the presence of submicellar NaDC and NaC concentrations induces dye dimerization and the extent of dimerization is higher in NaDC than in NaC [46]. At concentrations of bile salts higher than CMC, stable bile salts micelles are formed and mitoxantrone molecules are incorporated in the micelles in monomer form. The absorption maxima of mitoxantrone are red shifted in both NaTDC and NaTC micelles in comparison with pure aqueous medium. This red shift in the absorption maxima of mitoxantrone can be explained by the transfer of mitoxantrone molecules from the highly polar phase (phosphate buffer) into a less polar phase (the hydrophobic micelles) and point toward the existence of strong interactions between mitoxantrone monomers and bile salts micelles.

### 2.2. Determination of Binding Constant, Partition Coefficient and Thermodynamic Parameters

**Figure 10:**
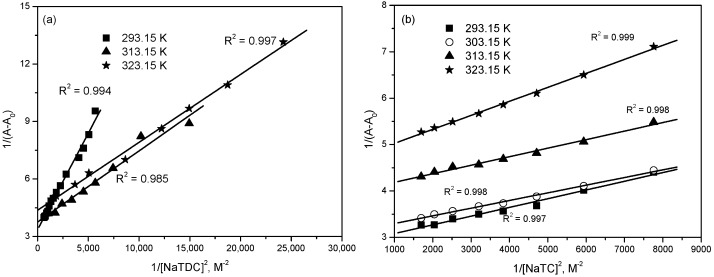
Benesi-Hildebrand plots for the binding interaction of mitoxantrone with (**a**) NaTDC and (**b**) NaTC micelles at different temperatures.

The mitoxantrone absorption changes at 660 nm in the presence of varying bile salts concentrations were used to estimate the binding constant of mitoxantrone to bile salts micelles and the partition coefficient of mitoxantrone between aqueous and micellar phases, and the respective thermodynamic parameters. The binding constant (K_b_) and the stoichiometric ratio (n) were estimated from the double reciprocal Benesi-Hildebrand plots of 1/(A-A_0_) versus 1/[BS]^n^ (Equation (4)). These plots should be linear for the correct stoichiometry (n) [50]. As shown in Figure 10, a linear Benesi-Hildebrand plots were obtained for mitoxantrone–bile salts micelle binding at all investigated temperatures when n = 2 indicating a 1:2 stoichiometry for these complexes. The results are presented in Table 1 for both bile salts.

The binding constant values reveal that mitoxantrone binds stronger to NaTDC micelles than NaTC micelles for all investigated temperatures. This result can be explained by the difference in the hydrophobicity of micelles core of the two bile salts. NaTDC and NaTC have the same head group but the presence of an additional hydroxyl group in NaTC structure leads to a more hydrophilic surface of NaTC than in the case of NaTDC. The observation suggests that hydrophobic interactions between mitoxantrone monomers and NaTDC micelles are much stronger compared to that between mitoxantrone monomers and NaTC micelles. Also, the binding constant increases with increasing temperatures for both bile salts.

**Figure 11:**
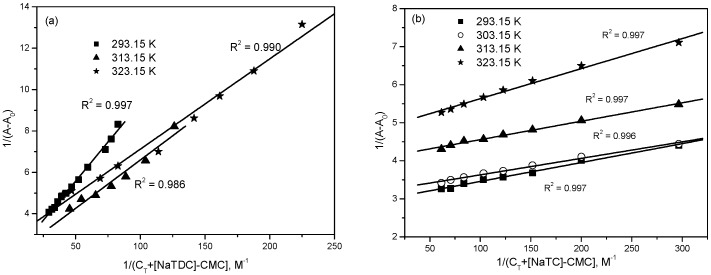
Plot of 1/(A-A_0_) versus 1/(C_T_ + [BS]-CMC) for the interaction of mitoxantrone with (**a**) NaTDC and (**b**) NaTC at different temperatures.

Partition coefficient (K_x_) is an important thermodynamic parameter used to assess the distribution of drug molecules between the micellar phase and the bulk aqueous phase. The value of K_x_ is obtained from the slope of the plot of 1/∆A versus 1/(C_T_ + [BS] − CMC) (Equation (5)) as shown in Figure 11 for different temperatures. The results are summarized in Table 2.

The analysis of the results from Table 2 indicates that mitoxantrone shows quite high partition coefficients for both bile salt micelles but higher for NaTC in comparison with NaTDC for all investigated temperatures. Therefore, it is concluded that mitoxantrone monomers are entrapped more efficient in NaTC micelles and this difference may be due to the presence of an extra hydroxyl group in NaTC which make it more hydrophilic. Also, the results show that the partition coefficient increases with the increase in temperature for both bile salts.

The values of the Gibbs free energy of interaction ($\Delta G_{b}^{0}$) and the Gibbs free energy of the transfer of drug from bulk aqueous phase to micellar phase ($\Delta G_{x}^{0}$) and the corresponding standard enthalpy (∆H^0^) and the standard entropy (∆S^0^) changes were calculated from the values obtained for K_b_ and K_x_ at different temperatures from the spectral studies using the following equations:

(1) $$\Delta G^{0}=-\mathrm{RTlnK}$$

(2) $$\Delta H^{0}=\frac{\partial (\Delta G^{0}/T)}{\partial (1/T)}$$

(3) $$\Delta S^{0}=\frac{\Delta H^{0}-\Delta G^{0}}{T}$$

**Figure 12:**
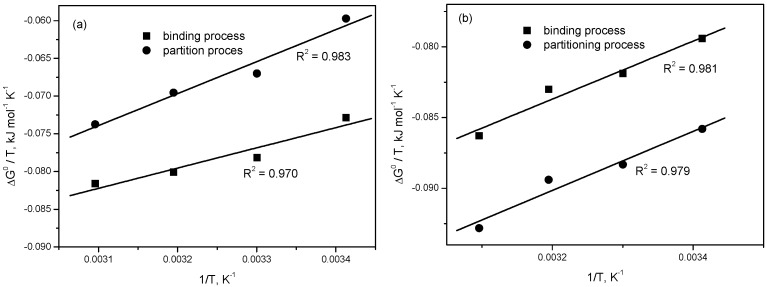
Plot of ∆G^0^/T versus 1/T for the binding and partition of mitoxantrone to (**a**) NaTDCand (**b**) NaTC.

According to Equation (2), the plot of ∆G^0^/T versus 1/T is linear and the slope of this plot is equal with ∆H^0^ (Figure 12).

As seen from Table 1 and Table 2, ∆G^0^ values are negative at each investigated temperature for both binding and partitioning processes of mitoxantrone to NaTDC and NaTC micelles indicating the spontaneity of both processes. Moreover, the ∆G^0^ values becomes more negative with the increase of temperature for both processes meaning that the binding and partitioning of mitoxantrone to the bile salts micelles are preferred at higher temperatures. The positive values of ∆H^0^ indicate that both binding and partitioning processes are endothermic. The net ∆H^0^ is the sum of the change in enthalpies arising from hydrophobic interactions, electrostatic interactions and hydration of polar head groups. The positive values of ∆S^0^ and ∆H^0^ indicate that the binding and partitioning processes of mitoxantrone to both NaTDC and NaTC micelles are entropy controlled over the range of studied temperatures. The difference in the entropy changes could be a reflection of the changes in water accessible surface area when the complex is formed. When the water is transferred from the hydration shell of a non-polar molecule to the bulk a strong positive contribution to ∆S is observed [51,52]. Therefore, the positive values of ∆S^0^ and ∆H^0^ indicate that the hydrophobic interactions play a major role in the binding and partition processes of mitoxantrone with NaTDC and NaTC micelles. Furthermore, the higher positive values of ∆H^0^ and ∆S^0^ for NaTDC micelles could reflect a more pronounced hydrophobic interaction, in agreement with higher hydrophobicity of NaTDC than NaTC. Thermodynamic parameters presented in Table 1 and Table 2 reflect distinct types of mitoxantrone interaction with NaTDC and NaTC micelles supported by the differences in nature and structure of bile salts micelles. In NaTC micelles the molecules associate with their hydrophobic sides (back-to-back association), while in NaTDC micelles hydrophobic and hydrophilic sides face each other (back-to-face association) due to the less hydrophilic face and the closer contact this arrangement allows for the slightly curved ring system [53,54].

### 2.3. EPR Studies on Mitoxantrone/Micellar Bile Salts Systems

EPR spectroscopy represents a valuable tool in investigating the non-homogeneity at micro scale level of a given system. In this study we choose three amphiphilic spin probes from the family of doxyl type spin probes based on their suitability to investigate micellar systems in general [41] and in the case of bile salts, in particular [55]. The paramagnetic moieties attached to the stearic acid backbone in different positions allow analysis of the polarity and dynamic of paramagnetic molecules in the bile salts micelles as a function of the structural particularities of the systems components.

**Figure 13:**
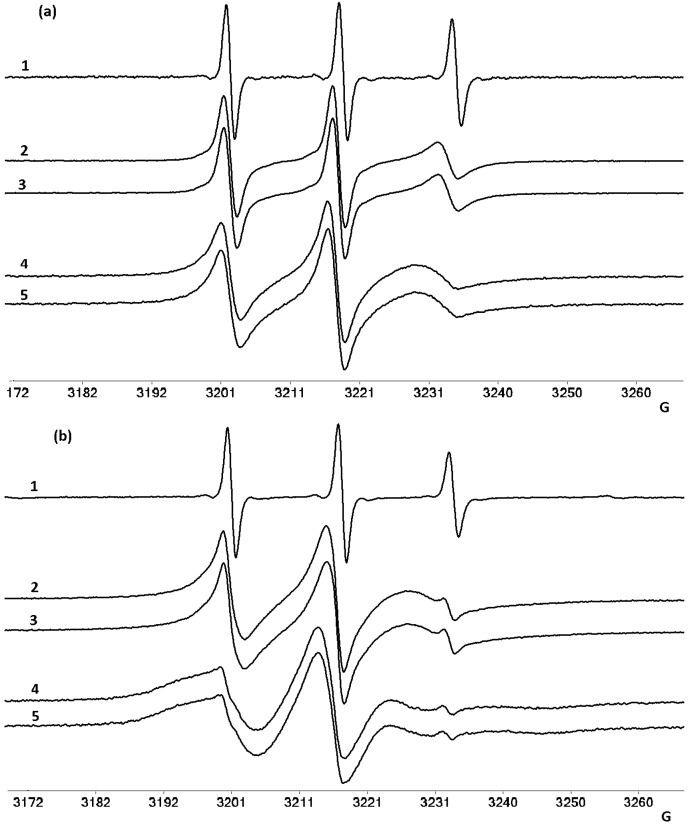

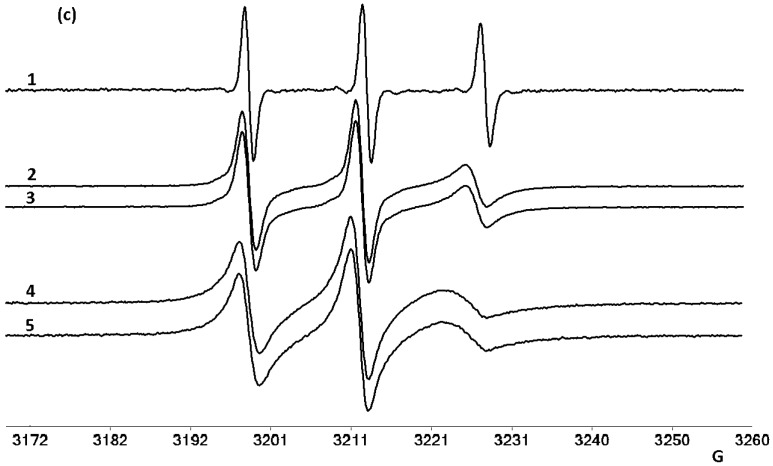
EPR spectra of 5-DSA (**a**); 12-DSA (**b**) and 16-DSA (**c**) in the following solutions: phosphate buffer (1), NaTC micelles (2) NATC and mitoxantrone (3), NaTDC micelles (4) and NaTDC and mitoxantrone (5).

In Figure 13 are presented the EPR spectra of 5-, 12- and 16-(4,4-dimethyl-3-oxazolidinyloxy) (abbreviated as 5-DSA, 12-DSA and 16-DSA, respectively) spin probes in micelles of NaTDC and NaTC (1.00 × 10^−2^ M) in the absence and the presence of mitoxantrone. It can be observed that in phosphate buffer solution all three spin probes have the same nitrogen coupling constant (a_N_) value (15.85 G) corresponding to a hydrophilic environment. At the same time, all spin probes exhibit an isotropic motion (spectra 1 in Figure 13a–c).

The values of rotational correlation time of the 5-DSA, 12-DSA and 16-DSA, in micellar solutions of NaTDC and NaTC alone and in the presence of mitoxantrone are presented in Table 3.

The molar ratio bile salts/spin probe is 100:1 and concentration of each bile salt is 10 to 25 times higher than CMC. Therefore, we assume that the presence of spin probe on the micellar size can be neglected and the dynamic of the equilibrium between bile salts monomers and micelles is not perturbed. In micellar solutions of bile salts used in the present study, the spin probes exhibit significant changes of their EPR parameters. The a_N_ values of spin probes dissolved in micellar solutions decrease as the nitroxide moiety senses a more hydrophobic environment. The values of a_N_ for 5-DSA and 16-DSA indicate that the paramagnetic moiety senses a more hydrophobic environment in both type of micelles than 12-DSA. For example, a_N_ corresponding to EPR spectra of 5-DSA and 16-DSA is 15.25 G in NaTC micelle solution and 14.9 G in NaTDC micelles solution. 12-DSA senses a less hydrophobic environment as the values of a_N_ are 15.5 G and 15.65 G in NaTC and NaTDC respectively. These observations reveal that at microscale level, the polarity inside the micelles is not uniform. In both micellar solutions it was noticed a slower motion of the spin probes compared to the buffer solution which also indicate the formation of micelle/spin probe complex. Also, the τ values for all spin probes are higher for NaTDC micelles than that for NaTC micelles, indicating a difference in properties of these micelles.

While in the case of 5-DSA and 16-DSA the rotational correlation time reveals a weak immobilization of the spin probes (Figure 13a,c), in the case of 12-DSA the spin probe is moderately immobilized (Figure 13b). In the same time, the EPR spectra of 12-DSA in micellar solutions exhibit a two component spectra (Figure 13b, spectra 2–5). One component corresponds to a fast motion while the other one shows a moderately immobilization. The structures of spin probes used in this study are linear and flexible and beside the amphiphilic character, these aspects are relevant for orientation of paramagnetic moiety attached to the backbone in a given environment. Likely, the stearic chain adopts a bended conformation, which in our case determines orientation of nitroxide moiety toward different microenvironments. The results suggest that in the case of 12-DSA, the nitroxide is oriented close to the interface micelle/water which explain the higher values of a_N_ observed in this case. Moreover, in all cases it was noticed that NaTC micelle is more hydrophilic than NaTDC micelle.

The EPR spectra of these spin probe also can give indirect information on the orientation of the mitoxantrone in bile salts micelles, by comparison of rotation correlation time observed in the absence and in the presence of the drug. Mitoxantrone concentration is lower than of spin probe, therefore it is normal do not expect an exclusion of the spin probe from the micelle. On the other hand at the mitoxantrone concentration used in this study, the drug will not influence the assembly of the bile salts. Although the dynamic regime of the spin probe is not influenced by the presence of mitoxantrone, the rotational correlation time increases. In the case of 5-DSA the variation of this parameter is larger and this suggest that locally the environment is more viscous due to the presence of mitoxantrone.

## 3. Materials and Methods

### 3.1. Materials

Mitoxantrone dihydrochloride, NaTC, NaTDC and the spin probes 5-DSA, 12-DSA and 16-DSA were analytical grade and supplied by Sigma Aldrich (St. Louis, MO, USA). All the compounds were used without further purification. Experiments were performed in 0.1 M phosphate buffer (pH 7.4) and 0.1 M carbonate buffer (pH 10) and deionized water (18.2 MΩcm, Mili-Q water purification system) was used for the preparation of solutions.

### 3.2. UV-Vis Absorption Spectroscopy

The absorption spectra of pure mitoxantrone and in the presence of different concentrations of NaTDC and NaDC bile salts were recorded in the wavelength range of 400–800 nm and different temperatures (293.15 K, 303.15 K, 313.15 K and 323.15 K) by using a JASCO V-630 spectrophotometer equipped with a Peltier-controlled ETCR-762 model accessory (JASCO Corporation, Tokyo, Japan). The measurements were done using a matched pair of quartz cuvettes of 1.0 cm optical length. The absorption titration experiments were performed by successive additions of concentrated bile salts stock solution directly into a cuvette containing 2 mL mitoxantrone solution. After addition of bile salt aliquots, the mixtures were shaken well and the absorption spectra were registered after equilibration. The binding constants of mitoxantrone to bile salts and micelle-water partition coefficients were determined from the monomer absorbances of series of solutions containing a fixed drug concentration and increasing bile salts concentrations.

The binding constant (K_b_) and the stoichiometric ratio (n) for mitoxantrone-bile salts complexes were estimated from the Benesi-Hildebrand equation [56,57]:

(4) $$\frac{1}{A-A_{0}}=\frac{1}{K_{b}\left(A_{1}-A_{0}\right){\left[BS\right]}^{n}}+\frac{1}{A_{1}-A_{0}}$$

where [BS] is the concentration of bile salts, A, A_0_ and A_1_ are the absorbance values of mitoxantrone in presence of bile salt, in absence of bile salt and the absorbance due to the formation of drug-bile salt complex.

Partition coefficient (K_x_) was evaluated from the following equation, according to the pseudo-phase model [58,59]:

(5) $$\frac{1}{\Delta A}=\frac{1}{\Delta A_{\infty }}+\frac{n_{W}}{K_{x}\Delta A_{\infty }\left(\left[BS\right]+C_{T}-\mathrm{CMC}\right)}$$

where ∆A = A − A_0_, ∆A_∞_ = A_1_ − A_0_, C_T_ is the total drug concentration and n_w_ = 55.5 M is the molarity of water.

The visible absorption spectra of mitoxantrone alone and in the presence of different bile salts concentrations were deconvoluted using Gaussian multi-peaks function in PeakFit 4.11 software (Systat Software Inc., Chicago, IL, USA), in order to assess the evolution of the overlapping spectral bands. Linear fitting of the experimental data was performed using Origin 7.0 software (MicroCal Software, Inc., Piscataway, NJ, USA).

### 3.3. EPR Spectroscopy

The EPR spectra were recorded on a FA 100 spectrometer (JEOL, Tokyo, Japan) at room temperature, with the following parameters: frequency modulation of 100 kHz, microwave power of 0.998 mW, sweep time of 480 s, modulation amplitude of 1 G, time constant of 0.3 s, and magnetic field scan range of 100 G. The rotational correlation times of the spin probe showing an isotropic dynamic regime were determined using Equation (6):

(6) τc=6.51×10−10ΔH0[(h0h−1)12+(h0h+1)1/2−2]

where ΔH_0_ is the peak-to-peak width (in Gauss) of the central line, and *h*_−1_, *h*_0_ and *h*_+1_ are the heights of the low, central and high field lines, respectively [55].

The EPR spectra of spin probes incorporated into micelles exhibit a slow dynamic and in some cases a two component feature. In these cases the EPR spectral simulations were performed using the program developed by Budil et al., based on non-linear least-squares (NLSL) fits [60]. Stock solutions of spin probes (10^−2^ M) were prepared in ethanol. To prepare samples for EPR measurements, in each case, an appropriate volume of ethanol solution of the spin probe was evaporated from a vial under a stream of inert gas. Subsequently a buffer solution (containing NaTC, NaTDC or mitoxantrone) was added to reach a spin probe concentration of approximately 10^−4^ M. To record EPR spectrum, each solution was transferred to glass capillaries and sealed.

## 4. Conclusions

The interaction between anticancer drug mitoxantrone and NaTDC (a dihydroxy) and NaTC (a trihydroxy) bile salts has been investigated by combining UV-Vis absorption and EPR spectroscopy techniques. The results indicated that the presence of submicellar concentrations of both bile salts induces mitoxantrone aggregation, but NaTDC induces a higher aggregation of mitoxantrone (the higher aggregates are the major component in solution as against NaTC when monomer and dimer are major components in solution). For micellar bile salt concentrations, mitoxantrone monomers are entrapped in the micellar core. The binding of mitoxantrone is stronger for NaTDC micelles than NaTC micelles for all investigated temperatures and these results can be explained by the difference in the micellar structure as well as the hydrophobicity of two bile salts. The positive high values of both ∆S_b_^0^ and ∆H_b_^0^ are indicative of enhanced hydrophobic interactions between hydrophobic chromophore of mitoxantrone and hydrophobic surface of bile salts. The positive high values of both ∆H^0^ and ∆S^0^ indicate that the binding and partitioning processes of mitoxantrone to both NaTDC and NaTC micelles are entropy controlled and the hydrophobic interactions are the main interaction forces between mitoxantrone and both bile salts.

The analysis of the EPR spectra of amphiphilic spin probes from doxyl family demonstrated the no homogeneity of the bile salt micelles. Thus, it was proved that NaTC micelles represent a less hydrophobic environment compared with NaTDC micelles. In the same time the spin probe motions inside NaTDC are more restricted compared with NaTC. The rotational correlation time reflects dynamic of the spin probe in a given environment and the results shown that this parameter increases in the case of mitoxantrone/bile salt micelles.

Because the cell membrane is the first obstacle to be overcome by drugs in order to enter cells and micelles are known as simple model systems for complicated biological membranes, the present results represent a first step towards the general understanding of the drug-biomimicking structures interaction.

## Figures and Tables

**Table 1:** Binding constants and thermodynamic parameters for the interaction of mitoxantrone with NaTDC and NATC.

NaTDC					NaTC			
T (K)	K_b_/10^4^ (M^−2^)	$\Delta G_{b}^{0}$(kJ mol^−1^)	$\Delta H_{b}^{0}$(kJ mol^−1^)	$\Delta S_{b}^{0}$(J mol^−1^ K^−1^)	K_b_/10^3^ (M^−2^)	$\Delta G_{b}^{0}$(kJ mol^−1^)	$\Delta H_{b}^{0}$(kJ mol^−1^)	$\Delta S_{b}^{0}$(J mol^−1^ K^−1^)
293.15	0.64 ± 0.05	−21.35	26.85	164.42	1.41 ± 0.08	−23.27	20.49	149.27
303.15	1.21 ± 0.09	−23.68		166.68	1.89 ± 0.07	−24.81		149.43
313.15	1.52 ± 0.09	−25.06		165.77	2.17 ± 0.08	−25.98		148.40
323.15	1.83 ± 0.08	−26.36		164.66	3.21 ± 0.09	−27.87		149.65

**Table 2:** Partition coefficients and thermodynamic parameters for the interaction of mitoxantrone with NaTDC and NaTC in phosphate buffer pH 7.4.

	NaTDC				NaTC			
T (K)	K_x_/10^3^	$\Delta G_{x}^{0}$(kJ mol^−1^)	$\Delta H_{x}^{0}$(kJ mol^−1^)	$\Delta S_{x}^{0}$(J mol^−1^ K^−1^)	K_x_/10^4^	$\Delta G_{x}^{0}$(kJ mol^−1^)	$\Delta H_{x}^{0}$(kJ mol^−1^)	$\Delta S_{x}^{0}$(J mol^−1^ K^−1^)
293.15	1.32 ± 0.04	−17.50	42.39	204.30	3.03 ± 0.04	−25.14	20.87	156.95
303.15	3.16 ± 0.08	−20.30		206.80	4.10 ± 0.07	−26.76		157.11
313.15	4.29 ± 0.07	−21.77		204.89	4.67 ± 0.02	−27.98		156.00
323.15	7.11 ± 0.09	−23.82		204.89	7.06 ± 0.05	−29.98		157.36

**Table 3:** Rotational correlational time (τ) of the 5-DSA, 12-DSA and 16-DSA in micellar solutions of NaTDC and NaTC (2.00 × 10^−2^ M), alone and in the presence of mitoxantrone (2.55 × 10^−5^ M).

System	5-DSA	12-DSA		16-DSA
	τ_1_ (s)	τ_1_ (s)	τ_2_ (s)	τ_1_ (s)
Phosphate buffer, pH 7.4	2.55 × 10^−10^	2.50 × 10^−10^		1.23 × 10^−10^
NaTDC	2.9 × 10^−9^	5.63 × 10^−11^	6.39 × 10^−9^	2.02 × 10^−9^
mitoxantrone + NaTDC	3.50 × 10^−9^	7.32 × 10^−11^	6.72 × 10^−9^	2.44 × 10^−9^
NaTC	2.13 × 10^−9^	5.87 × 10^−11^	3.26 × 10^−9^	1.18 × 10^−9^
mitoxantrone + NaTC	2.56 × 10^−9^	3.85 × 10^−10^	3.44 × 10^−9^	1.28 × 10^−9^
